# Chronic cough and a normal chest X-ray - a simple systematic approach to exclude common causes before referral to secondary care: a retrospective cohort study

**DOI:** 10.1038/npjpcrm.2015.81

**Published:** 2016-03-03

**Authors:** Richard D Turner, Graham H Bothamley

**Affiliations:** 1 Department of Respiratory Medicine, Homerton University Hospital NHS Foundation Trust, London, UK; 2 Barts and The London School of Medicine and Dentistry, Queen Mary University of London, London, UK

## Abstract

Chronic cough is common in the community and can cause significant morbidity. It is not clear how closely treatment guidelines are used in general practice, or how often specialist referral is indicated. We aimed to assess the management of chronic cough in primary care before referral to a cough clinic, and to assess the outcome of managing chronic cough with an approach of simple investigation and empirical treatment trials. Data were extracted from the records of all patients attending a district general hospital respiratory clinic over a two-year period with isolated chronic cough lasting ⩾8 weeks. The clinic assessed symptoms with a cough-severity visual analogue scale and the Leicester Cough Questionnaire. Among 266 patients, the most frequent diagnoses were asthma (29%), gastro-oesophageal reflux (22%) and angiotensin-converting enzyme inhibitor use (14%). In all, 12% had unexplained chronic cough. Common diagnoses had often not been excluded in primary care: only 21% had undergone spirometry, 86% had undergone chest radiography and attempts to exclude asthma with corticosteroids had been made only in 39%. In the clinic few investigations were conducted that were not available in primary care. Substantial improvements in symptoms occurred with a median (interquartile range) total of 2 (2–3) clinic visits. We estimated that 87% of patients could have been managed solely in primary care; we did not identify distinguishing characteristics among this group. Most cases of chronic cough referred to secondary care could be managed with a simple and systematic approach, which is potentially transferrable to a community setting.

## Introduction

Cough is common in primary care.^1^ Although most coughs are short-lived and self-limiting, those that persist have an impact on the quality of life.^2^ By definition, isolated chronic cough lasts >8 weeks and is unexplained by chest X-ray findings.^3^ Smoking, angiotensin-converting enzyme inhibitor (ACEi) medication, asthma, gastro-oesophageal reflux disease (GORD) and upper airway pathology (chronic rhinosinusitis or post-nasal drip) are considered common contributing causes,^4^ but the relative frequency of each probably depends on the clinical setting. Primary-care referrals are under-represented in the literature, as most reports come from tertiary-referral cough clinics.^5^

There are benefits to the patient and the wider health system from treatment in primary rather than secondary care.^6^ For the management of chronic cough, UK and international guidelines exist,^4,7,8^ with suggestions that much could be done in the primary-care setting without the need for complex investigation.^4^ Indeed, an explicit management pathway for chronic cough involving minimal investigation has been advocated, although it has only been tested in a well-established tertiary-referral cough clinic.^9^

There is evidence that referral to secondary care for chronic cough occurs prematurely. One survey from Northumberland reported that only 31% of general practitioners (GPs) were aware of published guidelines for chronic cough,^10^ and studies from two regions of England reported that, contrary to the UK guidance,^4^ <75% of patients had a chest X-ray and <40% underwent spirometry or a trial of corticosteroid treatment to help exclude asthma in primary care before onward referral.^10,11^

The aims of the current study were to review general practice management of chronic cough in patients later referred to secondary care, and to describe outcomes (final diagnoses and resolution of symptoms) from managing chronic cough with an approach based on simple investigation and empirical treatment trials in a district general hospital.

## Results

### Patient characteristics and clinic follow-up

**Figure 1:**
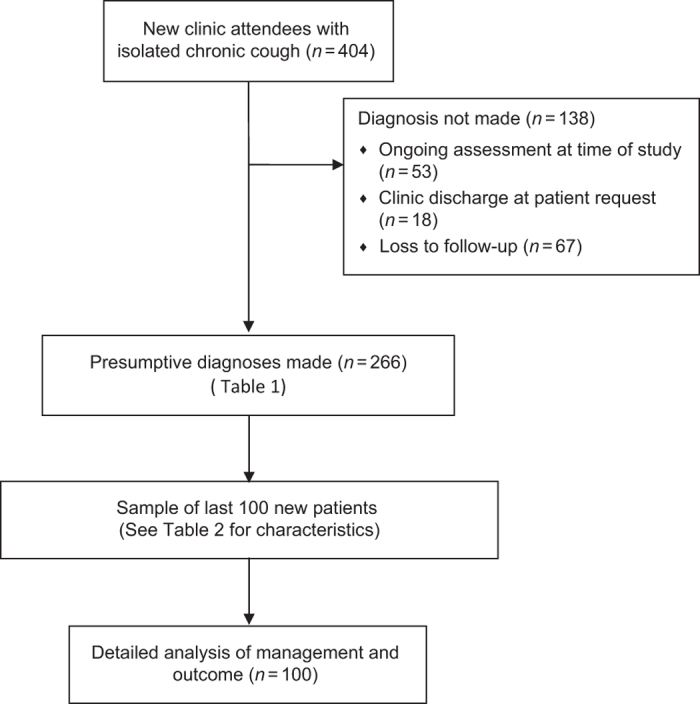
Flow of patients included in the study.

In total, 404 patients were referred with isolated chronic cough (Figure 1). Clinical records were available for all of them. More than 95% were seen by one clinician (RDT). The median (interquartile range (IQR)) age was 52 years (40–64), and 252 (62.4%) were female (*P*=0.001 for an expected equal sex ratio). Diagnoses were not made for 138 patients (34.2%), mainly because of ongoing assessment at the time of the study and loss to follow-up (Figure 1). In those who completed follow-up, the median (IQR) number of visits was 2 (2–3). Forty-five of the 67 patients (67%) who failed to attend a scheduled follow-up appointment attended the clinic only once. The previous clinic intervention in 20 of these 67 had been to prescribe a proton pump inhibitor (PPI), and in 25 to trial inhaled or oral corticosteroids. There were no differences in the duration of cough, cough severity or cough-related quality of life at the first clinic visit in all those who were lost to follow-up from those who completed their intended management (median (IQR) duration, 6 (3–12) vs 6 (3–18) months, *P*=0.98; Leicester Cough Questionnaire (LCQ) score, 10.2 (8.7–13.1) vs 9.4 (7.9–11.8), *P*=0.18; visual analogue scale (VAS) score, 63 (45–87) vs 72 (54–90), *P=*0.18, respectively).

### Diagnoses

Final diagnoses in the 266 patients who completed follow-up are given in Table 1, with asthma (28.7%), GORD (21.5%) and ACEi use (14.2%) being the most common. In all, 11.9% had unexplained chronic cough (UCC) and 19 (7.3%) had >1 diagnosis.

Of the 75 diagnosed with asthma, 23 (30.7%) had obstructive spirometry, of whom 14 (18.7% of all with asthma) demonstrated bronchodilator reversibility. In 46 of the 75 who had skin-prick tests, there was evidence for atopy in 24 (52.2%). Fourteen (58.3%) of those with atopy and an asthma diagnosis had normal spirometry. Thirty-seven (49.3%) were therefore diagnosed with asthma only because of a response to corticosteroids.

Of the 56 patients with presumed GORD-related cough, none underwent upper gastrointestinal studies from the clinic, although four (7.1%) had had previous investigations compatible with the diagnosis. Among the most recently discharged 100 patients, 19 responded to PPI treatment, including 10 of 31 reporting heartburn. Heartburn therefore had a sensitivity of 52.6% (95% confidence interval, 29.5–74.7%), a specificity of 74.1% (62.9–82.87%) and a positive predictive value of 32.2% (17.3–51.5%) for PPI-responsive cough.

The median (IQR) duration of symptoms in those assumed to have a post-infective cough was 3 (2–4) months. Sixteen of these 30 patients had other upper respiratory tract symptoms at the start of the prolonged cough, but the diagnosis was based on the self-limiting nature of symptoms. Four patients were diagnosed with lower respiratory tract infection (bronchitis) on the basis of symptoms and response to antibiotics.

Sixteen of the 17 patients with a diagnosis of upper airway-related cough (94%) had associated symptoms, although only four reported post-nasal drip. Other symptoms included nasal congestion, irritation and discharge, sneezing and hyposmia. Thirteen of the 17 were referred to the ear, nose and throat (ENT) clinic, of whom five subsequently had sinus computed tomography (CT). The diagnosis in four patients was made primarily on a response to nasal corticosteroids.

Two patients had subsequent diagnoses of malignancy: cancer of unknown primary involving the mediastinum, and metastatic prostate cancer. Both reported potentially concerning features on the first clinic visit (weight loss and previous prostate cancer, respectively) but had normal chest X-rays.

Of the 31 patients with UCC, 23 (74.2%) were female and the median age was 56 years (47–63), with symptoms for 12 (7–39) months at the first visit. This duration of symptoms was greater than in those given another diagnosis (6 (3–12) months, *P*<0.001). The median number of clinic visits in UCC was 3 (2–5). There was significant improvement in 16 of these 31 patients between visits despite not taking trials of treatment and having had the cough for a median of 12 (7–24) months at the first visit (compared with 12 (5–14) months in non-improving UCC; *P*=0.422). Symptoms were cyclical in six patients, of varying duration with no obvious underlying pattern.

### Primary-care management, clinic investigation and patient-reported outcomes

The characteristics of the last 100 patients to be discharged from clinic were similar to the entire group of all 404 clinic referrals, apart from in the smaller sample there were significantly more females and a higher proportion of diagnoses of asthma (Table 2). In all, 86 of the 100 had had a chest X-ray in primary care and only 21 had spirometry. Trials of treatment for cough had been antibiotics (in 64.0%), inhaled bronchodilators (53.0%), anti-GORD treatment (*n*=42; 32 PPI for >4 weeks), inhaled corticosteroids (36.0%), nasal steroids (25.0%), oral steroids (17.0%) and antihistamines (12.0%; Table 2). In all, 12.0% had been taking ACE inhibitors at the time, or within 2 months, of the first clinic visit; the medication had been stopped by the GP in five patients.

All 100 patients eventually had a chest X-ray and 97 had spirometry. Twenty-seven patients were referred to ENT; seven of these underwent sinus CT. Two were screened for tuberculosis. One had chest CT. None underwent bronchoscopy, bronchial hyper-reactivity testing or upper gastrointestinal studies. ENT assessment was perhaps unnecessary in 10 of the 27, as the underlying diagnosis was rhinosinusitis in only nine and unexplained cough in eight. In retrospect, there was a clear need for secondary care input in only 13% of the sample: nine diagnosed with rhinosinusitis following ENT assessment, one with bronchiectasis following CT and three with persistent unexplained cough.

**Figure 2:**
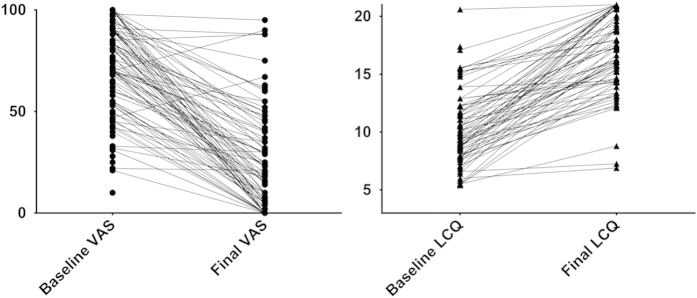
Patient-reported cough scores. Cough severity (VAS) and cough-related quality of life (LCQ) scores at initial and final clinic visits.

There were paired quality-of-life (LCQ) data for 76 of the 100 and paired cough-severity (VAS) scores for 96. Median (IQR) LCQ scores increased from 9.4 (7.9–11.8) at the first clinic visit to 17.3 (14.4–20.0) at discharge (*P*<0.001; maximum possible score 21). VAS scores decreased from 72 (54–90) to 23 (5–42; *P*<0.001; Figure 2). Changes in LCQ and VAS scores were correlated (Spearman’s *r*=−0.72, 95% confidence interval −0.82 to −0.58). In the five patients who showed no improvement in at least one of the scores, there was ongoing smoking (*n*=3), transfer to an asthma clinic (*n*=1) and a baseline LCQ score of near the maximum value, allowing little room for improvement (*n*=1). There was no association between final diagnosis and initial cough scores (data not shown). There was no evidence that patients who failed trials of empirical treatment (i.e., the 13 who would likely have not been treatable in primary care) had been more or less troubled by their cough at baseline than those who showed a response (VAS 81 (70–96) vs 70 (54–89), respectively, *P*=0.15; LCQ 9.2 (7.9–12.0) vs 9.7 (7.9–12.2), *P*=0.73), nor was there a difference in the duration of cough at the first clinic visit between the two groups (6 (3–20) vs 6 (3–12) months, respectively, *P*=0.76).

## Discussion

### Main findings

We demonstrated good patient outcomes in a secondary-care cough clinic over a median of only two clinic visits. Although ENT referral contributed to the diagnosis in 17%, we undertook very few investigations which were not available to GPs in the UK. Skin tests were commonly performed but were not essential for diagnosis. The majority (>80%) of cases of chronic cough referred from primary to secondary care could therefore be managed in a systematic and simple way.

Before referral from general practice, there had frequently been a failure to fully consider common causes of chronic cough by simple investigation (chest X-ray and spirometry) and interventions (empirical trials of treatment or withdrawal of an ACEi). The most common diagnoses were asthma, gastro-oesophageal reflux and ACEi use, together contributing to 65% of chronic coughs. Although those with coughs of a longer duration were more likely to have UCC, neither duration of cough nor other baseline characteristics that we investigated were associated with a subsequent failure of cough symptom scores to improve during follow-up.

### Interpretation of findings in relation to previously published work

Our findings are consistent with other reports from the United Kingdom,^10,11^ which indicate that referrals to secondary care for chronic cough are often premature (Table 2). This is in keeping with an observed low awareness of cough guidelines among GPs.^10^

For example, only a minority of patients had been adequately assessed for asthma, our most frequent diagnosis. All GPs should have access to spirometry,^12^ but only 21% of patients had undergone this investigation. Although often normal in asthma, spirometry may also pick up other diagnoses and is recommended by cough guidelines.^4^ There is only limited evidence for the effectiveness of bronchodilators in cough-variant asthma,^13^ yet this medication was tried by referring GPs in 53%. Because of frequent underlying eosinophilic airway inflammation, corticosteroids are the preferred treatment for cough-variant asthma,^4^ yet they were prescribed in just 39% before attending the clinic.

Similarly, we diagnosed ACEi-related cough in 14% presumably only because of an under-appreciation among the referring GPs of this phenomenon. ACE inhibitors can lead to chronic cough even months or years into therapy,^14^ probably by increasing cough reflex sensitivity to cause intolerance of otherwise innocuous stimuli.^15^ It is recommended that no-one presenting with chronic cough should continue ACEi treatment.^4^ ACEi-induced cough is straightforward to manage, although symptoms can take up to 3 months to resolve.^14^

Unlike in the current study, other cough clinics include referrals from secondary as well as primary care.^16–18^ Despite this, our diagnoses (amongst only primary care referrals) and their relative frequencies are similar to those reported elsewhere.^5^ The contributions of underlying diagnoses presumably vary with the sources of patient referral to cough clinics. We are unaware of any relevant reports but, because of the levels of specialist knowledge, ACEi- or asthma-related cough is probably referred from other respiratory clinics less frequently than from general practice. We estimated that unexplained cough and rhinosinusitis would be the most common diagnoses among GP referrals if a simple management algorithm had already been followed. Although this has yet to be tested directly, final diagnoses among a group of primary- and secondary-care referrals not responding to a similar algorithm in another clinic were idiopathic cough (39%), GORD (33%) and rhinitis (12%), with only one patient (6%) being diagnosed with asthma.^9^ If allowing for different referral sources and the fact that GORD as a major cause of cough is debatable,^19^ these findings are not dissimilar to our own.

The age and gender profile in our clinic was similar to that in other cough clinics,^20^ and patients with UCC were particularly likely to be female. This is consistent with a proposed gender difference in the mechanisms of cough,^20,21^ although women are more likely than men to seek medical attention for many symptoms.^22^

Because the clinic used few investigations, our diagnostic criteria differ from those in other studies.^23,24^ For example, we diagnosed cough-variant asthma primarily on response to corticosteroids without testing for bronchial hyperresponsivity or airway eosinophilia,^24^ and GORD on response to PPIs without corroboratory evidence from gastrointestinal studies.^19^ The influence of the extent of investigation on the final diagnosis is also problematic for the terms ‘unexplained’ or ‘idiopathic chronic cough’.^25,26^ For this reason, ‘cough hypersensitivity syndrome’ might be preferred to describe excessive coughing with or without a probable contributory diagnosis.^27,28^

The proportion of chronic cough attributed to rhinosinusitis in other series is highly variable,^5^ and the importance of ENT disease in chronic cough has been questioned.^13^ In keeping with this, our findings suggest that upper airway pathology is unlikely to be contributory in the absence of relevant symptoms. Imaging of the sinuses is likely to have low specificity in chronic cough^29^ and was not performed in our clinic.

Malignancy was the diagnosis in only two cases of chronic cough and normal chest X-ray, but assessment in the clinic led to early confirmatory investigations, prompted by features other than cough. This suggests that lung cancer is a rare diagnosis in patients with cough as the only symptom, a normal chest X-ray and no additional concerning features including smoking. Hence, CT is recommended in isolated chronic cough only after failure of other interventions.^4^

True habit or psychogenic cough exists but appears to be rare in adults.^30^ Correspondingly, we encountered this diagnosis in only 1.1%. Post-infective coughs generally resolve in <8 weeks^31^ but can potentially last longer.^4^ We made this diagnosis where symptoms were self-limiting, particularly with a history of acute upper respiratory tract symptoms. The longest duration of post-infective cough was 5 months, although it could have perhaps been better classed as UCC.^32^ As seen here, chronic cough can resolve spontaneously,^33^ and it may have done so more frequently if no treatment had been given, again indicating the difficulty in making diagnoses for chronic cough based on responses to medication.

Ojoo *et al.* ^9^ have also shown that the majority with chronic cough (72%) can be managed with minimum investigation. However, unlike in the current study, at least some of the treating physicians were experienced cough specialists, and patients were referred from secondary as well as primary care. Our findings therefore complement this other study, but they are more relevant to general practice. We are not aware of any other work that has attempted to identify baseline clinical characteristics of isolated chronic cough associated with a failure to respond to sequential empirical treatment trials.

### Strengths and limitations of this study

This is one of the largest surveys of the management of chronic cough, with final diagnoses for 266 patients. Furthermore, unlike many similar studies,^34^ we used validated patient-reported outcome measures to quantify responses to treatment.

A limitation of the study is our inability to comment on how chronic cough is managed in the community generally. As we have only observed primary-care management of chronic cough in those patients subsequently referred to the clinic, we do not know how GPs’ approach to cough varies. Although this does not affect our conclusion that the majority of chronic coughs should be treatable in primary care, observing variation in practice could lead to strategies for change. Our clinic conformed closely to the national British Thoracic Society guidelines on the management of chronic cough, and all patients in this cohort were seen by one clinician (RDT) on at least one occasion. Although this suggests consistency, we cannot comment on the use of our approach by a broader number of individuals, including in settings other than an inner-city population. However, our clinic algorithm was simple, and a very similar management strategy has shown similar success in Hull, where there is representation from both urban and rural settings.^9^

A substantial proportion of our cohort (>20%) was lost to follow-up. Although this is a potential shortcoming as we cannot verify the effectiveness of our interventions in these patients, we assumed that at least some of them improved precisely because they did not return for further advice.^4^ There were some missing data for VAS and LCQ scores, but insufficient numbers to affect the overall observed improvements in these values.

### Implications for future research, policy and practice

Further research would corroborate our findings in other settings, particularly to directly evaluate a similar approach to chronic cough in primary care. More generally, to overcome the inherent flaws in diagnosis by symptomatic response to treatment, an increased understanding of phenotypes of chronic cough is required through increased measurement of clinical variables.^35^

**Figure 3:**
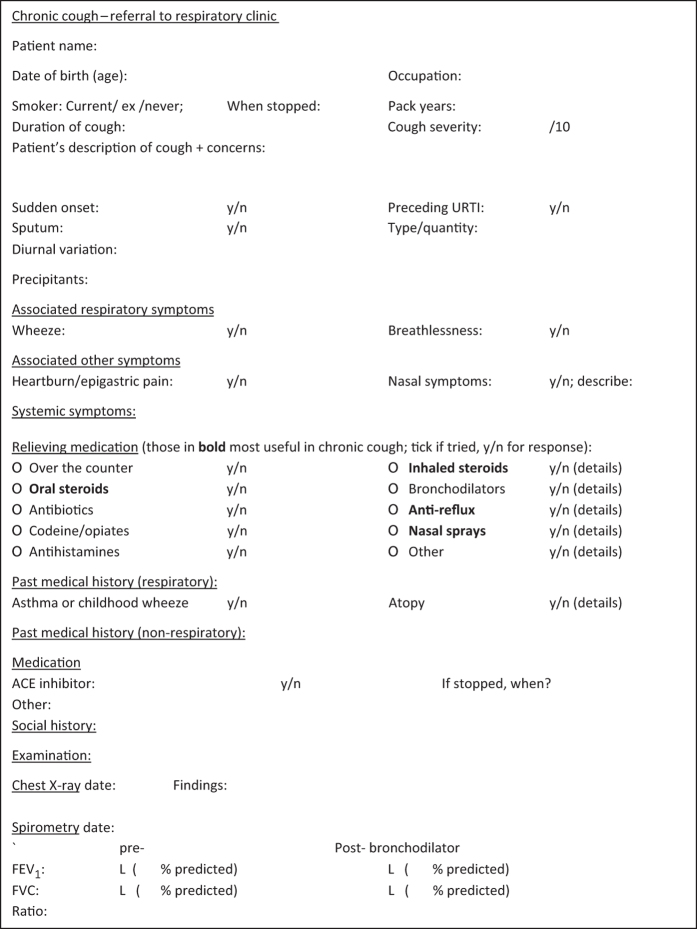
Example referral template to secondary care for chronic cough. URTI, upper respiratory tract infection. Adapted from ref. 4.

Specialist respiratory clinics will continue to have a role in chronic cough, for managing complicated cases and providing reassurance, but cough could be often managed more extensively in primary care before referral onwards. This should result in quicker resolution of symptoms and lower expenditure. Rather than necessarily seeing patients with chronic cough relatively early on, hospital physicians could suggest that GPs work through a standard referral template first. This could be based on the questionnaire published with the British Thoracic Society guideline^4^ and include information about chest X-ray and spirometry findings, and outcomes from withdrawing any ACEi medication and empirical treatment trials (Figure 3).

## Conclusions

Fewer patients with chronic cough could be referred to secondary care. The majority of patients in our clinic were managed successfully with a simple and systematic approach adaptable to general practice.

## Materials and methods

### Setting

**Figure 4:**
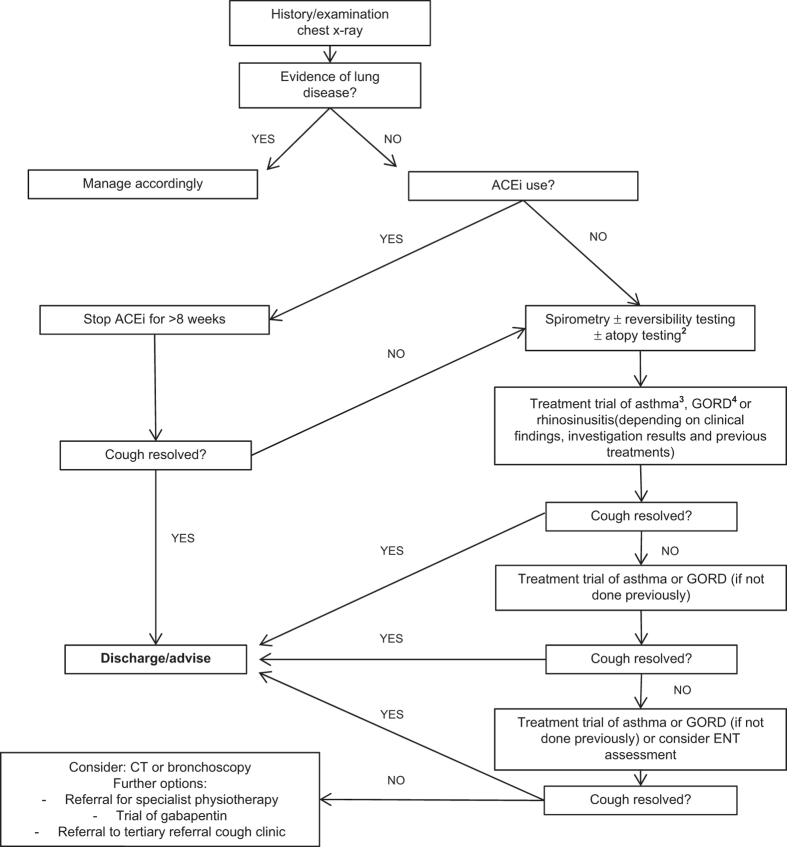
Algorithm for the management of chronic cough at Homerton Hospital cough clinic. 1, obstructive spirometry was followed by reversibility testing (positive if there was >12% increase in baseline forced expiratory volume in the first second (FEV_1_) with bronchodilator); 2, skin-prick tests to common aeroallergens supported a diagnosis of allergic asthma, although they are not specifically mentioned in UK cough guidelines (see ref. 4); 3, inhaled beclometasone (100–200 μg twice daily), or, if there was doubt about the inhaler technique or adherence to previous inhaled corticosteroid, a 10- to 14-day course of 30 mg daily prednisolone was considered; 4, high dose proton pump inhibitor, e.g., lansoprazole 30 mg or omeprazole 40 mg twice daily, even in the absence of dyspeptic symptoms; 5, trials of nasal steroids were generally only used in the presence of upper airway symptoms, or evidence of rhinitis or sinusitis on assessment in the ENT clinic.

This was a retrospective cohort study conducted at Homerton University Hospital, an inner-city district general hospital in London, UK. We set up a clinic in the respiratory department for GP referrals in July 2012 for the management of isolated chronic cough. Patients in whom GPs had a significant suspicion of cancer were referred to a separate clinic. The approach of the cough clinic followed that advocated by British Thoracic Society guidelines,^4^ with emphasis on 8- to 12-week trials of treatment before detailed investigation,^9^ as summarised in Figure 4. As the last step in the algorithm, options for persistent cough of unknown cause included referral to a respiratory physiotherapist for behavioural cough-suppression training,^36^ a trial of gabapentin^37^ or tertiary referral for entry into a clinical trial. For smokers, once serious pathology and airway disease were excluded, management was smoking cessation. From mid-2013, the clinic assessed patient-reported cough severity and cough-related quality at clinic visits with a 0–100 VAS^23^ and the LCQ,^38^ respectively.

### Data collection

Data were extracted from the records of all cough clinic attendees from July 2012 to July 2014. Patients were included if they been referred from general practice because of chronic cough as the only or predominant symptom of uncertain cause for ⩾8 weeks before the first visit. For all patients, age, gender, symptom duration, number of clinic attendances and final diagnosis were noted. A sample of the most recent 100 patients to complete follow-up was used to describe management undertaken before and after clinic referral, and patient-reported outcomes. Ethics committee approval was not sought, as the study was a review of the management of patients for whom we had clinical responsibility.

The minimum important difference for the VAS and LCQ was taken as 17 mm^39^ and 1.3,^40^ respectively. Data were analysed using Prism Version 6.04 (GraphPad Software Inc., San Diego, CA, USA), and non-parametric summary statistics (median and IQR) are reported as appropriate. Two-sided Mann–Whitney and Wilcoxon matched-pairs signed rank tests were used for comparing unpaired and paired data, respectively.

## Figures and Tables

**Table 1:** Final diagnoses of patients completing follow-up at Homerton Hospital cough clinic (*n*=266)

*Diagnosis*	n *(%)*
Asthma	75 (28.7)
Gastro-oesophageal reflux	56 (21.5)
ACEi use	37 (14.2)
Post-infective	30 (11.5)
Smoking	23 (8.8)
Upper airway pathology (rhinosinusitis)	17 (6.5)
COPD	5 (1.9)
Lower respiratory tract infection	4 (1.5)
Voluntary coughing/throat clearing	3 (1.1)
Malignancy	2 (0.8)
Bronchiectasis	1 (0.4)
Pulmonary fibrosis	1 (0.4)
Unexplained chronic cough	31 (11.9)
Spontaneously resolving	16 (6.1)
Persistent	15 (5.7)

Note: 19 patients (7.3%) had >1 diagnosis.

Abbreviations: ACEi, angiotensin-converting enzyme inhibitor; COPD, chronic obstructive pulmonary disease.

**Table 2:** Age and gender profile of referrals to cough clinic and management undertaken in primary care

*Location*	*Current study*			*Harding* et al.^11^	*Mackley* et al.^10^
	*East London, UK*			*South London, UK*	*Northumberland, UK*
*n*	404	100	*P*	66	47
Age	52 (40–64)	52 (40–66)	0.954	55 (15)	59 (27–84)
Gender (% female)	62	73	0.048	62	63
Duration of symptoms (months; median, range)	6 (2–216)	6 (2–120)	0.548	NA	7 (2–420)

*Final diagnosis (%)*^a^					
Asthma	28.7	39.0	<0.001	NA	NA
GORD	21.5	20.0	0.159		
ACEi	14.2	11.0	0.570		
Prior CXR (%)	NA	86		52	75
Prior spirometry (%)	NA	21		17	39
Trial of antireflux treatment (%)	NA	32		17	50
Trial of inhaled or oral steroid treatment	NA	39		35	NA
Trial of nasal steroid	NA	25		NA	NA
Trial of antibiotic	NA	64		NA	NA
Trial of inhaled bronchodilator	NA	53		NA	NA

Whole group compared with a smaller sample in the current study (Figure 1) and in context of other work. Values are median (IQR) or mean (s.d.), unless otherwise stated.

Abbreviations: ACEi, angiotensin-converting enzyme inhibitor; CXR, chest X-ray; GORD, gastro-oesophageal reflux disease; NA, not available.

a Diagnoses only made for 266 of initial 404 patients; Figure 1.

## References

[bib1] Morice, A. H. Epidemiology of cough. Pulm. Pharmacol. Ther. 15, 253–259 (2002). 12099774 10.1006/pupt.2002.0352

[bib2] Birring, S. S. et al. Cough frequency, cough sensitivity and health status in patients with chronic cough. Respir. Med. 100, 1105–1109 (2006). 16266801 10.1016/j.rmed.2005.09.023

[bib3] Pratter, M. R. , Brightling, C. E. , Boulet, L. P. & Irwin, R. S. An empiric integrative approach to the management of cough: ACCP evidence-based clinical practice guidelines. Chest 129, 222S–231S (2006). 16428715 10.1378/chest.129.1_suppl.222S

[bib4] Morice, A. H. , McGarvey, L. & Pavord, I. Recommendations for the management of cough in adults. Thorax 61 (Suppl 1): i1–i24 (2006). 16936230 10.1136/thx.2006.065144 PMC2080754

[bib5] Chung, K. F. & Pavord, I. D. Prevalence, pathogenesis, and causes of chronic cough. Lancet 371, 1364–1374 (2008). 18424325 10.1016/S0140-6736(08)60595-4

[bib6] NHS England, Public Health England, Health Education England, Monitor, Care Quality Commission, NHS Trust Development Authority. Five year forward view (2014). Available at www.england.nhs.uk/wp-content/uploads/2014/10/5yfv-web.pdf (accessed 4 March 2015).

[bib7] Morice, A. H. et al. The diagnosis and management of chronic cough. Eur. Resp. J. 24, 481–492 (2004). 10.1183/09031936.04.00027804 15358710

[bib8] Irwin, R. S. , French, C. T. , Lewis, S. Z. , Diekemper, R. L. & Gold, P. M. Overview of the management of cough: CHEST guideline and expert panel report. Chest 146, 885–889 (2014). 25080295 10.1378/chest.14-1485 PMC4694189

[bib9] Ojoo, J. C. et al. Management of patients with chronic cough using a clinical protocol: a prospective observational study. Cough 9, 2 (2013). 23347748 10.1186/1745-9974-9-2 PMC3565860

[bib10] Mackley, R. , Schatzberger, T. & Parker, S. Management of chronic cough in primary care [abstract]. Thorax 68, A20 (2013).

[bib11] Harding, R. et al. Primary care management of chronic cough [abstract]. Eur. Resp. J. 42, 683s (2013).

[bib12] Levy, M. L. et al. Diagnostic spirometry in primary care: proposed standards for general practice compliant with American Thoracic Society and European Respiratory Society recommendations. Prim. Care Respir. J. 18, 130–147 (2009). 19684995 10.4104/pcrj.2009.00054 PMC6619276

[bib13] Birring, S. S. Controversies in the evaluation and management of chronic cough. Am. J. Respir. Crit. Care Med. 183, 708–715 (2011). 21148722 10.1164/rccm.201007-1017CI

[bib14] Dicpinigaitis, P. V. Angiotensin-converting enzyme inhibitor-induced cough: ACCP evidence-based clinical practice guidelines. Chest 129, 169S–173S (2006). 16428706 10.1378/chest.129.1_suppl.169S

[bib15] Morice, A. H. , Lowry, R. , Brown, M. J. & Higenbottam, T. Angiotensin-converting enzyme and the cough reflex. Lancet 2, 1116–1118 (1987). 2890021 10.1016/s0140-6736(87)91547-9

[bib16] Kastelik, J. A. et al. Investigation and management of chronic cough using a probability-based algorithm. Eur. Resp. J. 25, 235–243 (2005). 10.1183/09031936.05.00140803 15684286

[bib17] Birring, S. S. et al. Chronic tonsillar enlargement and cough: preliminary evidence of a novel and treatable cause of chronic cough. Eur. Resp. J. 23, 199–201 (2004). 10.1183/09031936.03.00066403 14979491

[bib18] O’Connell, F. , Thomas, V. E. , Pride, N. B. & Fuller, R. W. Capsaicin cough sensitivity decreases with successful treatment of chronic cough. Am. J. Respir. Crit. Care Med. 150, 374–380 (1994). 8049818 10.1164/ajrccm.150.2.8049818

[bib19] Kahrilas, P. J. , Smith, J. A. & Dicpinigaitis, P. V. A causal relationship between cough and gastroesophageal reflux disease (GERD) has been established: a pro/con debate. Lung 192, 39–46 (2014). 24221340 10.1007/s00408-013-9528-7 PMC4118765

[bib20] Morice, A. H. et al. A worldwide survey of chronic cough: a manifestation of enhanced somatosensory response. Eur. Resp. J. 44, 1149–1155 (2014). 10.1183/09031936.00217813 25186267

[bib21] Kelsall, A. , Decalmer, S. , McGuinness, K. , Woodcock, A. & Smith, J. A. Sex differences and predictors of objective cough frequency in chronic cough. Thorax 64, 393–398 (2009). 19131447 10.1136/thx.2008.106237

[bib22] Green, C. A. & Pope, C. R. Gender, psychosocial factors and the use of medical services: a longitudinal analysis. Soc. Sci. Med. 48, 1363–1372 (1999). 10369437 10.1016/s0277-9536(98)00440-7

[bib23] McGarvey, L. P. A. et al. Evaluation and outcome of patients with chronic non-productive cough using a comprehensive diagnostic protocol. Thorax 53, 738–743 (1998). 10319055 10.1136/thx.53.9.738 PMC1745317

[bib24] Brightling, C. E. , Ward, R. , Goh, K. L. , Wardlaw, A. J. & Pavord, I. D. Eosinophilic bronchitis is an important cause of chronic cough. Am. J. Respir. Crit. Care Med. 160, 406–410 (1999). 10430705 10.1164/ajrccm.160.2.9810100

[bib25] Pratter, M. R. Unexplained (idiopathic) cough: ACCP evidence-based clinical practice guidelines. Chest 129, 220S–221S (2006). 16428714 10.1378/chest.129.1_suppl.220S

[bib26] McGarvey, L. P. A. Idiopathic chronic cough: a real disease or a failure of diagnosis? Cough 1, 9 (2005). 16270939 10.1186/1745-9974-1-9 PMC1277011

[bib27] Morice, A. H. et al. Expert opinion on the cough hypersensitivity syndrome in respiratory medicine. Eur. Resp. J. 44, 1132–1148 (2014). 10.1183/09031936.00218613 25142479

[bib28] Morice, A. H. The cough hypersensitivity syndrome: a novel paradigm for understanding cough. Lung 188 (Suppl): S87–S90 (2010). 19809853 10.1007/s00408-009-9185-z

[bib29] Hansen, A. G. et al. Incidental findings in MRI of the paranasal sinuses in adults: a population-based study (HUNT MRI). BMC Ear Nose Throat Disord. 14, 13 (2014). 25674037 10.1186/1472-6815-14-13 PMC4324827

[bib30] Irwin, R. S. , Glomb, W. B. & Chang, A. B. Habit cough, tic cough, and psychogenic cough in adult and pediatric populations: ACCP evidence-based clinical practice guidelines. Chest 129, 174S–179S (2006). 16428707 10.1378/chest.129.1_suppl.174S

[bib31] Braman, S. S. Postinfectious cough: ACCP evidence-based clinical practice guidelines. Chest 129, 138S–146S (2006). 16428703 10.1378/chest.129.1_suppl.138S

[bib32] Haque, R. A. , Usmani, O. S. & Barnes, P. J. Chronic idiopathic cough: a discrete clinical entity? Chest 127, 1710–1713 (2005). 15888850 10.1378/chest.127.5.1710

[bib33] Yousaf, N. , Montinero, W. , Birring, S. S. & Pavord, I. D. The long term outcome of patients with unexplained chronic cough. Respir. Med. 107, 408–412 (2013). 23261310 10.1016/j.rmed.2012.11.018

[bib34] French, C. T. , Diekemper, R. L. & Irwin, R. S. CHEST Expert Cough Panel. Assessment of intervention fidelity and recommendations for researchers conducting studies on the diagnosis and treatment of chronic cough in the adult: CHEST guideline and expert panel report. Chest 148, 32–54 (2015). 25764280 10.1378/chest.15-0164 PMC4493878

[bib35] Turner, R. D. & Bothamley, G. H. Cough hypersensitivity syndrome: clinical measurement is the key to progress. Eur. Resp. J. 45, 1507–1508 (2015). 10.1183/09031936.00212714 25931491

[bib36] Chamberlain, S. et al. Efficacy Of A Physiotherapy, Speech And Language Therapy Intervention (PSALTI) On Health Related Quality Of Life (HRQOL) for patients with refractory chronic cough: a randomised control trial [abstract]. Thorax 69, A78 (2014).

[bib37] Ryan, N. M. , Birring, S. S. & Gibson, P. G. Gabapentin for refractory chronic cough: a randomised, double-blind, placebo-controlled trial. Lancet 380, 1583–1589 (2012). 22951084 10.1016/S0140-6736(12)60776-4

[bib38] Birring, S. S. et al. Development of a symptom specific health status measure for patients with chronic cough: Leicester Cough Questionnaire (LCQ). Thorax 58, 339–343 (2003). 12668799 10.1136/thorax.58.4.339 PMC1746649

[bib39] Spinou, A. & Birring, S. S. An update on measurement and monitoring of cough: what are the important study endpoints? J. Thorac. Dis. 6, S728–S734 (2014). 25383207 10.3978/j.issn.2072-1439.2014.10.08 PMC4222923

[bib40] Raj, A. A. , Pavord, D. I. & Birring, S. S. Clinical cough IV:what is the minimal important difference for the Leicester Cough Questionnaire? Handb. Exp. Pharmacol. 187, 311–320 (2009). 18825348 10.1007/978-3-540-79842-2_16

